# Classification of Migraine Using Static Functional Connectivity Strength and Dynamic Functional Connectome Patterns: A Resting-State fMRI Study

**DOI:** 10.3390/brainsci13040596

**Published:** 2023-03-31

**Authors:** Weifang Nie, Weiming Zeng, Jiajun Yang, Le Zhao, Yuhu Shi

**Affiliations:** 1 Lab of Digital Image and Intelligent Computation, Shanghai Maritime University, Shanghai 201306, China; 2 Department of Neurology, Shanghai Sixth People’s Hospital, Shanghai Jiao Tong University School of Medicine, Shanghai 200233, China

**Keywords:** dynamic functional connectome, sliding window analysis, clustering, migraine, classification

## Abstract

Migraine is a common, chronic dysfunctional disease with recurrent headaches. Its etiology and pathogenesis have not been fully understood and there is a lack of objective diagnostic criteria and biomarkers. Meanwhile, resting-state functional magnetic resonance imaging (RS-fMRI) is increasingly being used in migraine research to classify and diagnose brain disorders. However, the RS-fMRI data is characterized by a large amount of data information and the difficulty of extracting high-dimensional features, which brings great challenges to relevant studies. In this paper, we proposed an automatic recognition framework based on static functional connectivity (sFC) strength features and dynamic functional connectome pattern (DFCP) features of migraine sufferers and normal control subjects, in which we firstly extracted sFC strength and DFCP features and then selected the optimal features using the recursive feature elimination based on the support vector machine (SVM−RFE) algorithm and, finally, trained and tested a classifier with the support vector machine (SVM) algorithm. In addition, we compared the classification performance of only using sFC strength features and DFCP features, respectively. The results showed that the DFCP features significantly outperformed sFC strength features in performance, which indicated that DFCP features had a significant advantage over sFC strength features in classification. In addition, the combination of sFC strength and DFCP features had the optimal performance, which demonstrated that the combination of both features could make full use of their advantage. The experimental results suggested the method had good performance in differentiating migraineurs and our proposed classification framework might be applicable for other mental disorders.

## 1. Introduction

As a common episodic disorder, migraine is clinically characterized by recurrent, mostly unilateral, moderate-to-severe pulsatile headache with symptoms such as nausea, vomiting, photophobia, and fear of sound [1,2]. The 1-year prevalence of migraine in the general population is 12% [3] and it is listed as the third most common disease and the second leading reason of disability by the World Health Organization [4], which seriously affects the daily life and learning work of patients. At present, the pathogenesis of migraine is very complex and is still in the stage of exploration. The diagnosis of migraine is mainly based on the patient’s family history, clinical manifestations, and combined with the third edition of the International Classification of Headache Diseases (ICHD-3) [2] for comprehensive judgment. The International Classification of Headache Diseases is constantly being updated to provide standardization for the diagnosis of migraine and guide clinicians in the assessment of patients. However, there is a lack of positive imaging signs for migraine diagnosis and for the time being there are no uniform imaging biomarkers and a lack of an objective imaging ‘gold standard’ that would quantify the components of the criteria [5]. Therefore, the study of objective imaging biomarkers for the diagnosis and classification of migraine and its various types is a new direction and a hot topic that will also help to further optimize the clinical diagnosis and treatment of migraine.

As a non-invasive method, resting-state functional magnetic resonance imaging (RS-fMRI) provides new ideas and exploratory perspectives for studying migraine diagnosis [6]. Based on RS-fMRI data, three features of low-frequency fluctuation amplitude, regional homogeneity, and regional functional correlation intensity were extracted and were used to differentiate migraine patients without aura from healthy controls together with regional gray matter volume features from structural MRI data and yielded a final classification accuracy of 83.67% [7]. Depending on the functional connectivity of 33 select ROI, the best classification accuracy of migraine was 86.1% using the 10-fold cross-validation method [8]. Combination deep learning methods and three functional measures (amplitude of low-frequency fluctuations, regional homogeneity, and regional functional correlation strength) of RS-fMRI data could distinguish not only between migraineurs and healthy controls but also between two subtypes of migraine [9]. One hundred and ninety-two resting-state FCs located primarily within the occipital lobe, the sensorimotor network, part of the medial-cerebellum, the cingulo-opercular network, the default mode network (DMN), and the frontal parietal network were identified and validated as neural markers of migraine without aura (MwoA), which could capture the unique features of MwoA and link changes in disease patterns to changes in the brain [10]. Meanwhile, the brain microstructure studies based on diffusion tensor imaging (DTI) have also provided structural insights into fMRI studies of the brain. Several migraine DTI studies had showed broader alterations in white matter tracts, subcortical and cortical areas, such as changes in the corpus callosum, thalamic radiations, coronal radiation, and the brainstem, which presented a high degree of variability during the migraine cycle phase [11,12,13,14]. DTI studies indicated that migraine had been linked to microstructural changes in a wide range of regions including the thalamic radiations, corpus callosum and brainstem which could accentuate neuronal damage and neuronal plasticity mechanisms [15]. All these studies suggested that resting-state functional magnetic resonance imaging could provide some objective imaging evidence for the diagnosis of migraine and assist clinicians at the imaging level in the management of the disease.

The studies mentioned above, however, were largely based on the assumption that brain connections and functions were static in the scan processing. This assumption ignored the fact that individuals may have different mental activities at different points in time [16,17]. Some studies had also shown that there were complex spatial and temporal changes during the task processing state as well as altered functional activity in the resting state in the brain [18,19]. As a result, there is also growing interest in exploring the information contained in the temporal characteristics of RS-fMRI data and capturing the fluctuating state of the brain during scanning. Static functional connectivity (sFC), static network functional connectivity (sFNC), and dynamic network functional connectivity(dFNC) of six resting-state networks were compared between migraine patients and normal control subjects to provide evidence that the functional features of the chronic migraine brain may fluctuate over time and it was concluded that the chronicity of migraine may be related to abnormal pattern connections between sensory and cognitive brain networks [20]. By adopting sliding window cross correlation, clustering state analysis, and graph-theory approach on dynamic functional connectivity (dFC) analysis, a transient pathologic state with atypical thalamo ortical connectivity in migraineurs was demonstrated [21]. Based on the Kmeans algorithm, the dFC patterns of 43 brain networks were studied and it was found that the functional connectivity of FPN, brainstem, and cerebellum showed significant intergroup differences [22]. A comparison of static functional connectivity and dynamic functional connectivity in 59 regions of interest (ROI)) revealed that migraine and persistent post-traumatic headache differed significantly in 17 regions in static functional connectivity and 10 regions in dynamic functional connectivity. There was overlap in ROIs, but not in the functional connectivity pairs [23]. By investigating the topological properties of the dFC among brain networks of migraineurs from a multichannel hierarchical perspective, the dFC and corresponding global topological properties between migraineurs and normal controls showed a significant difference, while local topological properties and dynamic fluctuations were susceptible to the effects of time window length [24].

The above studies have shown that migraine and normal individuals differ significantly at the group level, whether from a static global perspective or a dynamic local perspective. In this work, therefore, we used static functional connectivity (sFC) strength and dynamic functional connectome (DFCP) features, respectively, as well as a combination of both, to classify migraineurs from healthy subjects. We hypothesized that, as a neurological disorder, migraineurs had abnormalities in static and dynamic global brain function compared with normal subjects and these abnormalities could contribute to the clinical manifestations of migraineurs. The sFC strength features provided average connectivity information over overall time, whereas DFCP features were better at capturing the immediate fluctuating state of the brain. Therefore, we assumed that sFC and DFCP had complementary characteristics which could be combined for classification. To ensure the reliability and reproducibility of the conclusion, the test samples used in this paper to verify the results did not participate in features extraction by the 5-fold cross-validation method. For further validation of the reproducibility of the results, a second normal control group was used for testing in this paper and similar results were obtained. The results showed that the algorithm combining sFC and DFCP features had better classification performance. This further validated our hypothesis.

## 2. Materials and Methods

### 2.1. Data Acquisition

Raw RS-fMRI data from 34 migraineurs (19 males and 15 females; average age: 36.12 years [range: 17–58 years]) were obtained from the Department of Neurology of Shanghai Jiao Tong University Affiliated Sixth People’s Hospital before preprocessing. The study was approved by the Independent Ethics Committee of Shanghai Sixth People’s Hospital East Campus and the consent form was informed before all participants participated in the study. All migraine patients were diagnosed with chronic migraine based on *International Classification of Headache Disorders 3rd Edition* criteria [2]. Rs-fMRI data were acquired using a 3T scanner (Siemens, Erlangen, Germany). During the scan, subjects were awake and were instructed not to think and to remain still. The scanning parameters were as follows: slice number = 38 (covering all brain areas), repetition time (TR) = 3.0 s, and number of time points = 160. After matching for age and gender, and according to the exclusion criterion (see Section 2.2 for details), the RS-fMRI data of 34 normal control subjects (19 males and 15 females; average age: 36.2 years [range: 18–58 years]) were acquired from a free public database (accessed on 15 December 2020 at http://fcon_1000.projects.nitrc.org/indi/retro/cobre.html) and were released by the Center for Biomedical Research Excellence which were abbreviated as COBRE; the parameters for the scans were as follows: slice number = 33 (covering all brain areas) and TR = 2.0 s, number of time points = 140. Based on the unpaired *t*-test with Welch’s correction, the *p*-value for ages was 0.9895, which indicated there was no significant difference between normal control subjects and migraineurs and also meant the data from the normal control group were gender-matched and age-matched with migraineurs group. To verify the repeatability of the results, another RS-fMRI data of 34 normal control subjects (19 males and 15 females; average age: 36.18 years [range: 19–62 years]) were acquired from a free public database (accessed on 5 December 2020 at http://fcon_1000.projects.nitrc.org/fcpClassic/FcpTable.html) and were released by Alan C. Evans which were abbreviated as ICBM; the parameters for the scans were as follows: slice number = 23 (covering all brain areas), TR = 2.0 s, and number of time points = 128. Meanwhile, the *p*-value for ages was 0.9844 which showed this dataset was also gender matched and age matched with the migraineurs group. This control group was not involved in the feature extraction and selection process and was used purely as a test set.

### 2.2. Data Preprocessing

Data Processing Assistant for Resting-State fMRI [25] was used to preprocess the RS-fMRI data, which involved the following steps: (a) slice timing; (b) head motion correction; (c) spatial normalization; and (d) spatial smoothing. Before slice timing correction, the first 10 time points were removed to avoid T1 equilibration effects and the middle slice was used as the reference frame for the slice-timing correction. Sinc interpolation and 6° transformation were applied to eliminate temporal and spatial offsets, respectively. To minimize the impact of motion, data for which there was >2 mm displacement in any direction or head rotation >1.5° were discarded. Spatial normalization involved reslicing to 2 mm × 2 mm × 2 mm using an echo-planar imaging template released by the Montreal Neurological Institute. A Gaussian kernel of 6 mm was applied to smooth the data.

### 2.3. Static Function Connectivity(sFC) Strength

The static functional connectivity(sFC) for each subject was estimated from the time series using the Pearson correlation method. Additionally, the time series were extracted by averaging RS-fMRI signal values of each ROI on the Brainnetome Atlas [26] with 246 ROIs. The Pearson correlation using all time points was calculated to determine sFC, as shown below.

(1) $${Corr}_{i,j}=\frac{Cov({TS}_{i},{TS}_{j})}{\sqrt{D({TS}_{i})}\sqrt{D({TS}_{j})}}\;\mathrm{if}\;i\neq j\;{Corr}_{i,j}=0\;\mathrm{if}\;i=j$$

where ${TS}_{i}$ (1 ≤ *i* ≤ 246) is the mean time serial for *i*th ROI.

The sFC strength vector was obtained by adding all positive values and removing the negative values of *Corr* for the same ROI, as negative connections are somewhat controversial.

(2) $${sFCS}_{i}=\sum_{j=1}^{246}\left({Corr}_{i,j}>0\right)$$

(3) $$sFCSV=\left[\begin{array}{c} {sFCS}_{1}\\ {sFCS}_{2}\\ \vdots \\ {sFCS}_{i}\\ \vdots \\ {sFCS}_{246} \end{array}\right]$$

where 1 ≤ *j* ≤ 246 and 1 ≤ *i* ≤ 246.

### 2.4. Dynamic Functional Connectivity Strength

In this paper, the dynamic functional connectivity (dFC) was extracted using the sliding time window method [27]. To explore the effect of different window lengths on the performance, the window lengths in this paper were selected as 12 s, 24 s, 36 s, 48 s, and 60 s with the step length as 1. The classification process was performed based on different window lengths, all of which are illustrated by taking 12 s as an example. After the window length was selected, the extraction of dFC was performed on each subject based on the whole brain according to the Pearson correlation coefficient with the window lengths of 12 s and the step size of 1. After that, the dFC strength (dFCS) of the whole brain was calculated by adding all absolute values of dFC based on ROI as the reference point. Finally, the dFCSs were combined to form a dFCS matrix. The specific implementation could refer to the previous study [28].

### 2.5. Automatic Generation of WQCPs

In our previous research work [28], it can be concluded that the colors of the adjacent columns in the dFCS matrix have a similarity. Therefore, to reduce the computation and improve the performance, the dFCS matrix can be segmented based on the similarity in color of the adjacent rows. In previous work [29,30], segment points were located manually; our previous research work used the calculation of the Euclidean distance between adjacent rows to automatically segment the matrix for improving efficiency and reducing manual errors. After locating the segmentation points, several divisions were extracted by dividing the dFCS matrix; for each segment, it could obtain the whole-brain quasi-stable connectome pattern (WQCP) (246 × 1) vector based on time averaging. The detailed algorithm could be found in the previous research [28].

### 2.6. Classification Framework

The classification performance of sFC strength, DFCPs, and a combination of these two methods were evaluated. Our main focuses were to investigate the performance of all the proposed classification models. To simulate the actual use of the scene and to test the effectiveness and repeatability of these models, a 5-fold cross-validation method was used to identify training and test samples for the migraine and the COBRE groups prior to feature extraction, with the ICBM group being categorized separately as the test set. Furthermore, recursive feature elimination based on support vector machine (SVM−RFE) was brought in this paper for extracting reliable features. SVM−RFE is a high-performance feature selection method combining support vector machine and backward elimination procedure [31]. Because of its efficient performance and strong generalization, it is widely used in the studies of biological information processing and image processing. The idea of the algorithm is to obtain the optimal feature subset by eliminating the suboptimal features one by one under maximizing the feature association classification accuracy. The SVM−RFE method was operated once per cross-validation fold. Then a linear support vector machine (SVM) classifier was used to evaluate the classification performance.

#### 2.6.1. Static Functional Connectivity(sFC) Strength Approach

**Figure 1:**
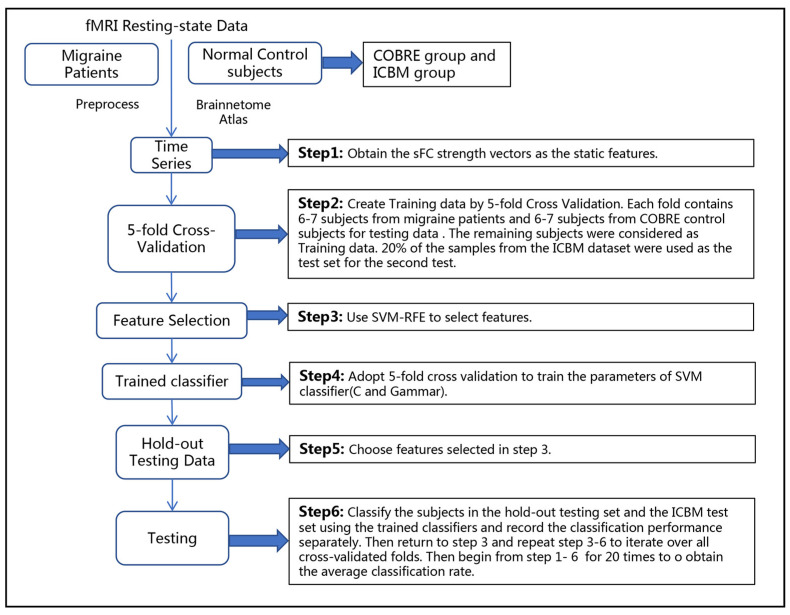
The framework of the classification approach based on sFC strength.

The sFC strength was obtained by adding all absolute values of sFC for the same ROI based on sFC matrix, which was a vector of 246 elements considered as the features. The SVM−RFE method was operated once per cross-validation fold to select the features. A 5-fold cross-validation strategy was used to estimate the generalization of this method for the migraine and the COBRE groups. Based on the features from training data, it was trained as an SVM classifier and then tested on hold-out testing samples from which the same features were selected, followed by a second test using the migraine group test set group and the ICBM group (see Figure 1 for the procedure of this method). Algorithm 1 gives the detailed steps of this method.
**Algorithm 1**: classification based on sFC strength featuresObtain sFC strength vectors for all the subjects using corresponding ROI time-courses as described in the Section 2.3.Determine the 5-fold cross-validation groups by first dividing data into 5 non-intersect folds, where each fold comprises 6 or 7 subjects from the healthy control group (COBRE group) and 6 or 7 subjects from the migraine group. One fold was considered as the hold-out testing set (13 or 14 testing subjects at each iteration). The remaining folds were the training set for each iteration. Meanwhile, 20% of the samples from the ICBM dataset were used as the test set for the second test. Note that, this step was the same in all classification algorithms.Use SVM−RFE to reduce the dimensionality reduction and select features.Based on the selected features train an SVM classifier with 5-fold cross-validation to select the best parameter.Build the testing set and select those selected features with the hold-out testing subjects in step 2.Classify the subjects in the hold-out testing set and the ICBM test set using the trained classifiers and record the classification performance separately. Return to step 3 and repeat steps 3–6 to iterate over all cross-validated folds. Then, begin from steps 1–6 20 times to obtain the average classification rate.

#### 2.6.2. Dynamic Functional Connectome Pattern (DFCP)Approach

**Figure 2:**
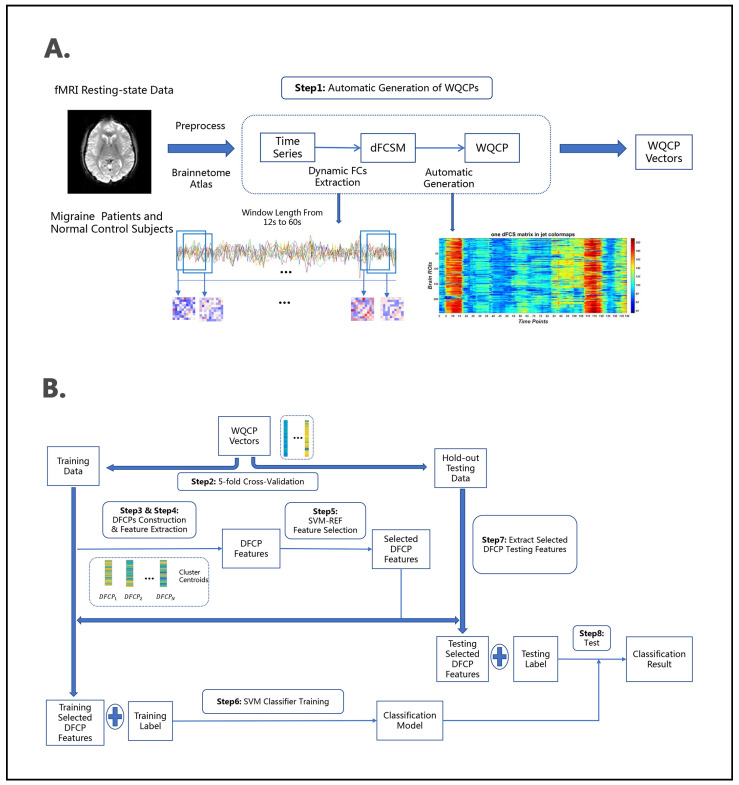
The framework of classification approach based on DFCP. (**A**) The preprocessed fMRI resting-state data were decomposed into 246 regions of interest (ROIs) by Brainnetome Atlas. The WQCP vectors were automatically generated from the resting-state functional connectivity (FCs) between the ROIs based on sliding time windows. (**B**) The WQCP vectors were clustered to obtain dynamic functional connectivity patterns and then the DFCP features were obtained. The DFCP features were sent into the feature selection and classification framework, which were performed on the basis of 5-fold cross-validation strategy. Both the migraine group and COBRE groups were divided into a training set and a test set, respectively, and ICBM group were used as test set. Selected features and SVM parameters were obtained from the migraine and COBRE training sets and were applied to the migraine and COBRE test sets. This process was repeated 100 times. This was followed by another 100 tests with the migraine and ICBM test sets. The final classification results were obtained for both sets of 100 times.

In our previous study [28], the auto-dynamic functional connectome model (A-DFCM) with twice clustering was presented to compare dynamic functional connectome patterns (DFCPs) from migraine patients and normal control subjects. Based on that study, we extracted the features based on dynamic functional connectome patterns using A-DFCM with twice clustering (see Figure 2 for illustration of the proposed model). First, the dFCS matrix was constructed using the sliding time window and WQCPs were built using an automatic segmentation algorithm. Then, 5-fold cross-validation was used to estimate the generalization error. In each cross-validation run, we performed twice-clustering (including K-means and hierarchical clustering) on the WQCPs from the training samples to obtain cluster states which were considered as the dynamic functional connectome patterns. Based on the DFCPs, the distribution ratio features and the mean regression coefficients could be extracted which is explained in detail later. SVM−RFE was applied to select reliable features once per cross-validation fold. Then, we used the selected features to train an SVM classifier and then tested on the held-out test samples. Algorithm 2 details the procedure of the approach.
**Algorithm 2**: classification based on DFCPs features1.Obtain WQCP vectors for all the subjects using A-DFCM.2.Determine the 5-fold cross-validation groups by dividing data into 5 non-intersect folds, where each fold comprises 6 or 7 subjects from the healthy control group (COBRE group) and 6 or 7 subjects from the migraine group. One fold was considered as the hold-out testing set (13 or 14 testing subjects at each iteration). The remaining folds were the training set for each iteration. Meanwhile, 20% of the samples from the ICBM dataset were used as the test set for the second test. Note that this step was the same in all classification algorithms.3.Obtain N cluster patterns by performing twice clustering (including K-means and hierarchical clustering) on the WQCPs from the training samples. Select the optimum number of cluster centroids per group (dynamic functional connectome patterns) based on the elbow criterion. There was one cluster centroid for each pattern which was set as *R_i_*, where *i* was the ith pattern.4.Obtain 2N DFCP features.
(a)Extract N features by calculating the distribution ratio of WQCP samples in each DFCP for each subject of training data as FeatRatio.
(4)$${Ratio}_{i}=\frac{\sum WQCP\;for\;Pattern\;i}{Sum(WQCP)}$$
(5)$$FeatRatio=\left[{Ratio}_{1},{Ratio}_{2},\ldots {Ratio}_{N}\right]$$(b)Note that each WQCP is assumed to be a linear combination of these patterns. Then, obtain the corresponding regression coefficient $\beta_{i}$ by regressing the WQCPs using the cluster centroids.
(6)$$WQCP=\beta_{1}*R_{1}+\beta_{2}*R_{2}+\cdots +\beta_{i}*R_{i}+\cdots +\beta_{N}*R_{N}$$
where $\beta_{i}$ (1 ≤ i ≤ N) is the ith corresponding regression coefficient for ith pattern.Then $\beta_{i}$ (1 ≤ i ≤ N) combined the regression coefficients β vector.
(7)$\beta V=\left[\beta_{1},\beta_{2},\ldots \beta_{N}\right]$(c)Calculate the mean regression coefficients mβV for all WQCPs of each subject resulting in the final N features. Then add features in step 4 a), consisting of the DFCP features (FeatDFCP) for the classification analysis.(8)$FeatDFCP=\left[FeatRatio\;m\beta V\right]$5.Extract reliable features by SVM−RFE.6.Using these selected FeatDFCP features, an SVM classifier was trained with 5-fold cross-validation on training data to select the best parameters (C and Gamma).7.With the hold-out subjects in step 2, extract and select the testing features as below:
(a)Decide the pattern of WQCP for each subject by calculating the distance between WQCP and each pattern centroid. The pattern with the minimum distance from WQCP is the pattern of WQCP.(9)$D_{i}=\sqrt{\sum_{j=1}^{j=246}{\left({wqcp}_{j}-r_{j}\right)}^{2}}$
(10)$Pattern\;of\;WQCP=\underset{1\leq i\leq N}{\min}D_{i}$(b)Calculate the distribution ratio of test subjects in each pattern.(c)Calculate the mean regression coefficients mβV for each subject as step 4.(d)Choose features selected.8.Classify the subjects in the hold-out testing set and the ICBM test set using the trained classifiers and record the classification performance separately. Then return to step 3 and repeat steps 3-8 to iterate over all cross-validated folds. Then begin from steps 2–8 20 times to obtain the average classification rate. Repeat steps 1-8 for each window length.

#### 2.6.3. Combined sFC Strength and DFCP Approach

**Figure 3:**
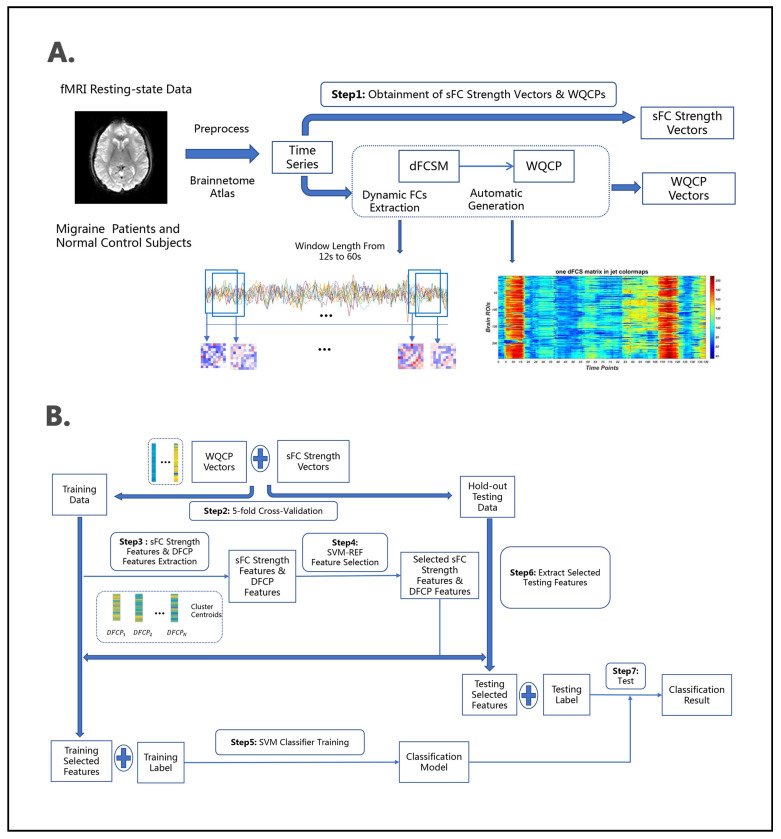
The framework of the classification approach based on combined sFC strength and DFCP methods. (**A**) The preprocessed fMRI resting-state data were decomposed into 246 regions of interest (ROIs) by Brainnetome Atlas. sFC strength vectors were obtained from the resting-state FC between the ROIs as mentioned in Algorithm 1. The WQCP vectors were automatically generated as mentioned in Algorithm 2. (**B**) The DFCP features were obtained based on WQCP vectors as mentioned in Algorithm 2. The sFC strength features and DFCP features were sent into the feature selection and classification framework, which were performed on the basis of 5-fold cross-validation strategy. Both the migraine group and COBRE groups were divided into a training set and a test set, respectively, and ICBM group were used as test set. Selected features and SVM parameters were obtained from the migraine and COBRE training sets and were applied to the migraine and COBRE test sets. This process was repeated 100 times. This was followed by another 100 tests with the migraine and ICBM test sets. The final classification results were obtained for both sets of 100 times.

For the combined sFC strength and DFCP approach, 246 features from sFC strength feature vector (obtained as mentioned in sFC strength approach section) and the DFCP features including distribution ratio features and the mean regression coefficients features (obtained as mentioned in the DFCP approach section) were used for classification. In addition, SVM−RFE, SVM classifiers, and a 5-fold cross-validation strategy were applied in a similar way as mentioned above (see Figure 3 for specific framework of the proposed model). Algorithm 3 describes the steps of the method in detail.
**Algorithm 3**: classification based on combined the sFC strength and DFCPs featuresObtain sFC strength vectors as mentioned in Algorithm 1 and obtain WQCP vectors as mentioned in Algorithm 2.Determine the 5-fold cross-validation groups by dividing data into 5 non-intersect folds, where each fold comprises 6 or 7 subjects from the healthy control group (COBRE group) and 6 or 7 subjects from the migraine group. One fold was considered as the hold-out testing set (13 or 14 testing subjects at each iteration). The remaining folds were the training set for each iteration. Meanwhile, 20% of the samples from the ICBM dataset were used as the test set for the second test. Note that this step was the same in all classification algorithms.Obtain 2N DFCP features as mentioned in Algorithm 2 and consider the sFC strength vectors as the static features.Extract reliable features by SVM−RFE.Using selected sFC strength features and DFCP features, a support vector machine (SVM) classifier was trained with 5-fold cross-validation on training data to select the best parameters (C and Gamma).With the hold-out subjects in step 2, extract and select the testing DFCP features as mentioned in Algorithm 2.Classify the subjects in the hold-out testing set and the ICBM test set using the trained classifiers and record the classification performance separately. Then, return to step 3 and repeat steps 3–7 to iterate over all cross-validated folds. Then, begin from steps 2–7 20 times to obtain the average classification rate. Repeat steps 1–7 for each window length.

### 2.7. sFC Strength Features

To further analyze the sFC strength features, we intersected all extracted feature sets to derive features with significant impact. Based on these features, key brain regions could eventually be obtained, details of which are given in Section 3.2.

### 2.8. DFCP Features

For DFCP features extraction, A-DFCM with twice-clustering model was used to identify dynamic functional connectome patterns. As the training samples for each classification process were different, the number of patterns of each classification was not the same as well. Therefore, the number of features per classification was not fixed. To estimate the DFCP features, we took ICMP dataset for the normal control group with the window length of 24 s which had the best classification performance in this dataset to present the detailed information in Section 3.3.

## 3. Results

### 3.1. Subsection

**Figure 4:**
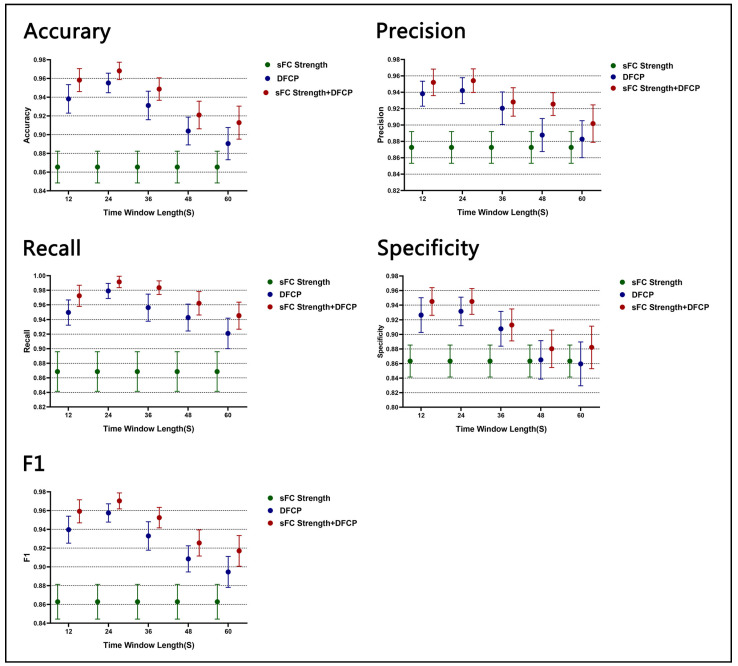
The classification accuracy, precision, specificity, recall, and F1 of sFC strength, DFCP, and sFC strength + DFCP approaches between the migraine patients and normal control (NC) subjects. On the *X*-axis, labels t = 12–60 indicate the time window length for DFCP, and *Y*-axis indicates the mean classification accuracy, precision, specificity, recall, and F1 on CI 95%.

A total of three classification approaches were evaluated in this study based on five classification performance metrics: accuracy, precision, recall, and F1. To illustrate the differences caused by different window lengths, we evaluated the DFCP method and sFC strength + DFCP combined method based on the window length from 12 s to 60 s using the COBRE dataset. The classification performance of the three methods is summarized and showed in Table 1, Figure 4, and Table 2. Table 2 shows the mean value, the standard deviation, and the mean with 95% CI of each classification performance metric under five window lengths for each classification approach. Figure 4 illustrates the mean value with 95% CI of accuracy, precision, recall, specificity, and F1 for all classification approaches. Table 2 displays the statistical *p*-values and significant differences of classification performance metrics along three approaches at the individual level under 5 different time window lengths, including accuracy, precision, recall, specificity, and F1. These comparison results were obtained by a two-sample *t*-test.

To illustrate the reliability and reproducibility of the three approaches, we computed the same classification performance metrics based on the ICBM test set with the window length from 12 s to 60 s. The mean value, the standard deviation, and the mean with 95% CI of each classification performance metric under five window lengths for each classification approach are displayed in Supplementary Table S1. The statistical *p*-values and significant differences in classification performance metrics along three approaches at the individual level under five different time window lengths, including accuracy, precision, recall, specificity, and F1, are showed in Supplementary Table S2. The mean values with 95% CI of accuracy, precision, recall, specificity, and F1 for all classification approaches are illustrated in Supplementary Figure S1.

### 3.2. sFC Strength Features Estimation

**Figure 5:**
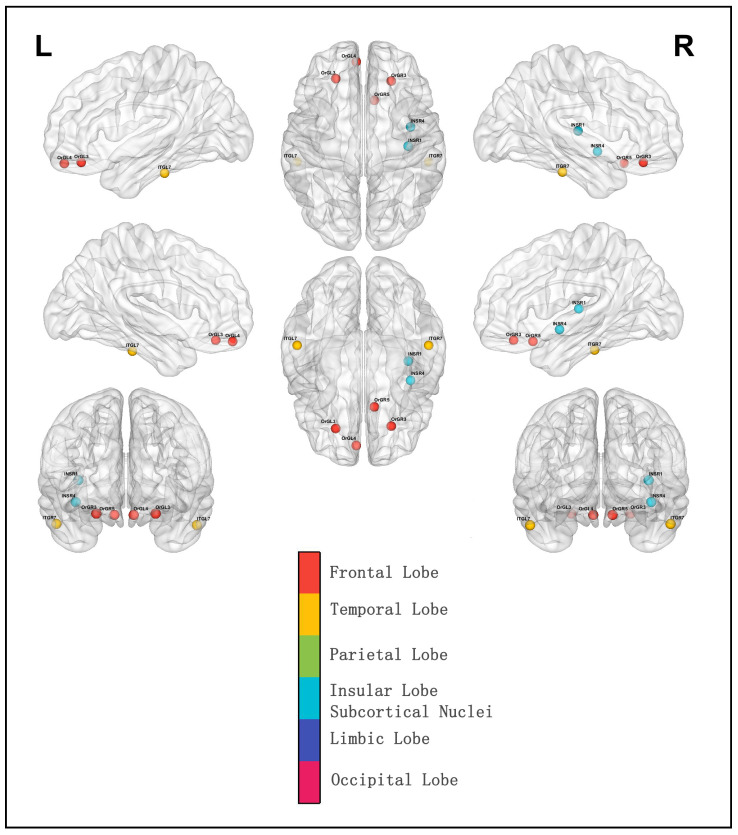
ROIs selected based on sFC strength feature intersection projected onto a standard brain surface.

The sFC strength feature were extracted based on the COBRE dataset according to the approach in Section 2.6.1. The critical eight brain ROIs were obtained by the method in Section 2.7. Table 3 shows the eight ROIs detailed information and Figure 5 shows the ROI projected onto a standard brain surface.

### 3.3. DFCP Features Estimation

**Figure 6:**
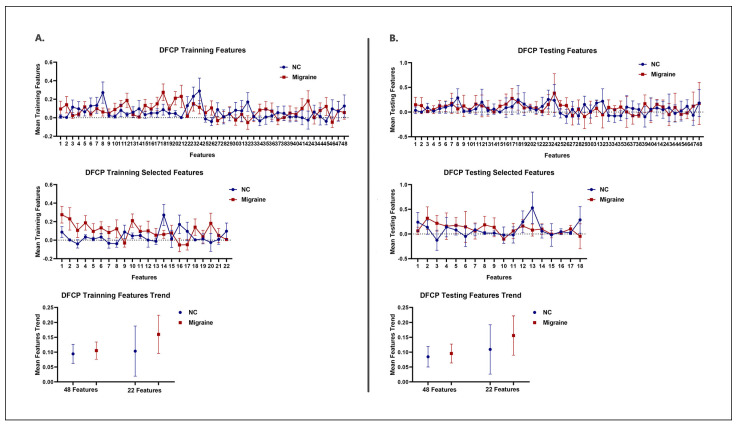
Features for DFCP classification approach. (**A**) The top picture shows mean training DFCP features with CI of 95% for two groups in all DFCP feature states. The middle picture displays mean training DFCP features with CI 95% for two groups in the selected DFCP feature states after SVM−RFE algorithm. The line presents the changing trend of features in two plots. The bottom picture shows the mean value with CI of 95% of the line in the top picture and the middle picture. (**B**) The top picture shows mean testing DFCP features with CI 95% for two groups in all DFCP feature states. The middle picture displays mean testing DFCP features with CI 95% for two groups in the selected DFCP feature states after SVM−RFE algorithm. The line presents the changing trend of features in two plots. The bottom picture shows the mean value with CI of 95% of the line in the top picture and the middle picture.

The DFCP features were now presented based on the best classification performance of which the time window length was 24 s using the COBRE dataset. In this classification process, 28 of migraine and 27 of normal people were the training sample and the rest were the testing sample. For the training sample, there were 1009 WQCPs, while for the testing sample, there were 250 WQCPs. Using twice clustering (including K-means and hierarchical clustering), 24 DFCPs were extracted; then, 24 distribution ratio features and 24 mean regression coefficient features were obtained, correspondingly. Then, using the SVM−RFE algorithm, 22 features were selected from all DFCP features. Figure 6 shows the training and testing DFCP features. In Figure 6A, the interleaved symbols plot showing group-wise mean training features (the ratio features and the mean beta coefficients) with CI 95% for all DFCP features in two training groups have been presented in the top, the same values for selected features with SVM−RFE have been displayed in the middle, while the line presented the changing trend of features in two plots. Then, the bottom plots show the mean value with CI 95% for the trend of all features and the selected features between the two training groups. Figure 6B shows similar information for DFCP features in the testing data. Further, Figure S2 shows the mean *p* value with CI 95% of 48 DFCP features between NC and migraine in the training set, the mean *p* value with CI 95% of 22 DFCP features between NC and migraine in the training set, and the same value in the test set.

## 4. Discussion

In this study, novel classification approaches of migraine based upon sFC strength and DFCP were presented to divided migraine patients from normal control individuals. To compare classification performance, only sFC strength features and DFCP features were used to classify the two groups. To explore the effect of the time window length on the classification results, the time window lengths in the dynamic function connection were from 12 s to 60 s.

### 4.1. Classification Performance

In our classification framework based on the COBRE dataset as the normal control group, the overall mean accuracy, precision, recall, specificity, and F1 of the sFC strength approach were 0.8653, 0.8726, 0.8686, 0.8633, and 0.8628, while the confidence interval was [0.8486 0.8821], [0.8535 0.8917], [0.8417 0.8955], [0.8416 0.8850], and [0.8461 0.8795]. For DFCP, the time window length 24s had the best performance with a mean accuracy, precision, recall, specificity, and F1 of 0.9552, 0.9420, 0.9790, 0.9314, and 0.9575; meanwhile, the confidence interval was [0.9449 0.9655], [0.9264 0.9576], [0.9688 0.9893], [0.9122 0.9507], and [0.9471 0.9678]. For the sFC strength + DFCP method, the best performance was also for time window length 24s, where the mean accuracy was 0.9681 with the confidence interval [0.9590 0.9773], the mean precision is 0.9541 the confidence interval [0.9399 0.9684], the mean recall is 0.9914 the confidence interval [0.9837 0.9992], the mean specificity is 0. 0.9450 the confidence interval [0.9275 0.9625], and the mean F1 is 0.9703 the confidence interval [0.9612 0.9795]. For other time window lengths, the detailed performance can be seen from Table 1.

The results from Table 1 and Figure 4 suggested that the approaches using DFCP and static FC strength + DFCP features performs better than the approach using sFC strength features for classification. Taking accuracy as an example, the mean accuracy of sFC strength approach was 0.8653 with the confidence interval [0.8486 0.8821], while DFCP was 0.8904 with the confidence interval [0.8734 0.9074] and sFC strength + DFCP was 0.9128 with the confidence interval [0.8954 0.9303] on the time window of 60s which were the minimal accuracy from 12 s to 60s, respectively. Furthermore, the mean accuracy of the DFCP approach was 0.9552 with the confidence interval [0.9449 0.9655], while sFC strength + DFCP was 0.9681 with the confidence interval [0.9590 0.9773] on the time window of 24 s which were the best accuracy, respectively. Other classification metrics (precision, recall, specificity, F1) also showed that both DFCP and sFC strength + DFCP outperformed sFC strength metrics. Furthermore, from the result, it also could display that sFC strength + DFCP features offered better performance than DFCP features.

From a statistical analysis perspective, it could be seen from Table 2 that classification based on DFCP and sFC strength + DFCP features significantly outperforms classification based on sFC strength features with the time window length of 12 s, 24 s, and 36 s for classification metrics. Meanwhile, for time window length 48 s and 60 s, the classification metrics including accuracy, recall, and F1 showed there were statistically significant differences between sFC strength and DFCP and the classification metrics including accuracy, precision, recall, and F1 had significant differences between sFC strength and sFC strength + DFCP. Additionally, between DFCP and sFC strength + DFCP, only the accuracy and F1 of 12 s, the recall of 24 s, and the recall and F1 of 36 s had significant differences.

Consistent conclusions could also be obtained from the results of the ICBM database calculation. From Tables S1 and S2 and Figure S1, the performance of the algorithm using DFCP features was higher than that of the algorithm using sFC strength features, while the algorithm using DFCP and static FC strength + DFCP features outperforms the algorithm using sFC strength features and the algorithm using DFCP features.

Based on the above results and analysis, sFC strength + DFCP features had the best classification performance, followed by DFCP features and sFC strength which had the worst. This showed that using sFC strength and DFCP features we can reliably discriminate migraines and normal controls at the individual subject level. That may occur because dynamic correlation features offered the information of local functional connectivity information for small time scales, so it likely reflected changes in macro neural activity patterns in terms of important aspects of cognition and behavior [32] and may capture critical information missing in static SFC strength methods [17]. In addition, the combination of sFC strength + DFCP features meant that both local information and global information are taken into account, which made the classification effect the best. Moreover, it also could be concluded that dynamicity-related features may be very useful in improving the classification performance, which had important implications in the exploration of migraines.

### 4.2. Time Window Length of DFCPs

Different time window lengths could have different effects on dynamic functional connectivity. If possible, to form the estimation of FC robust and to deal with the lowest frequencies of the signal, it should be large enough of the window. Meanwhile, to identify potentially interesting transients it should be small enough [32,33]. To explore which time window lengths were most appropriate for the algorithm and to investigate the reproducibility and reliability of the algorithm, five different time window lengths were used for experiments in this paper. From Table 4, it could be seen that there is a strong negative correlation between the time window length and the classification performance metric for DFCP features and sFC strength + DFCP features, as all the absolute values of correlation coefficients were greater than 0.7. For DFCP features and sFC strength + DFCP features, performance started to decrease as the time window length was extended to a certain length. From Table 2 and Figure 4, it also could be concluded that the best performance was achieved at 24 s, began to decrease at 36 s, and the worst performance was achieved at 60 s. Additionally, as seen in Table S1 and Figure S1, the performance was best at 24 s, started to decrease at 36 s, and was worst at 60 s. This may be because when the time window length was in a certain smaller range, it contained more dynamics and accommodated more subtle changes. This also implied that time window length variations may introduce more interesting information when exploring the neural mechanisms of migraine.

### 4.3. sFC Strength Features

In the classification method using sFC strength, the key eight ROIs that appeared in the feature set for each classification were mainly distributed in Brodmann area 11 and Brodmann area 13 in orbital gyrus of the frontal lobe; Brodmann area 20 in inferior temporal gyrus of the temporal lobe; and hypergranular insula, ventral dysgranular, and granular insula in insular gyrus of the insular lobe.

Note that the prefrontal cortex (PFC) and insular were part of the pain matrix which had information flow and integration in these areas [34,35,36,37,38]. In addition, it was found that the crucial cause of the significant decrease in reaction time during cognitive set shifting in migraine patients may be the abnormality of the frontal and parietal lobe [39]. Moreover, the temporal lobe and the occipital lobe had been implicated in migraine symptoms. Thus, it could be included that these eight ROIs were not only distributed in pain-related brain areas but also in the areas related to migraine symptoms, which might be the reason the features could play an important role in classification.

### 4.4. DFCP Strength Features

The DFCP features extracted by the algorithm SVM−RFE enabled better differentiation between migraine and normal individuals. From Figure 6A, we could see the detailed information and the trends of DFCP features between NC and migraine. Moreover, it could be displayed that differences in feature trends between NC and migraine in selected DFCP features station using SVM−RFE algorithm was greater than in all DFCP features station from the middle and bottom plots. Moreover, the *p*-value between the NC mean line and the migraine mean line was 0.6334 for all features and was 0.3497 for selected features which also implied the features were more diverse in selected features. Similar results in the testing set were found and shown in Figure 6B. Here, the *p*-value between the NC mean line and the migraine mean line was 0.6219 for all features and was 0.3970 for selected features. In Figure S2, the mean *p*-value of 48 DFCP features between NC and migraine in the training set is 0.205, while the confidence interval was [0.124 0.286]; the mean *p*-value of 22 DFCP features between NC and migraine in the training set is 0.082, while the confidence interval was [0.015 0.149]; the mean *p*-value of 22 DFCP features between NC and migraine in the training set is 0.331, while the confidence interval was [0.252 0.409]; the mean *p*-value of 22 DFCP features between NC and migraine in the training set is 0.306, while the confidence interval was [0.182 0.429]. From the distribution of the *p* value, it could also be concluded that the selected features were more discriminatory between migraine and normal individuals than all features.

### 4.5. Limitations and Future Directions

There were some limitations of this study. Firstly, the migraine and normal control data used in this paper were from different sequences/scanners as no normal control data could be acquired from the hospital due to the reason of reality. To try to avoid the effect of different data sources, this paper used the normal control data which were gender matched and age matched with the migraineurs group. Meanwhile, to avoid the effect of TR inconsistency the same time window length was taken to extract dynamic functional connectivity. Nevertheless, how to further reduce barriers to analysis due to data heterogeneities among different data sources would be an area where more effort needs to be invested in the future. Secondly, this paper adopted a fixed time window length to obtain dynamic functional connectivity. Although the performance of different time window lengths from 12 s to 60 s was investigated, the time window length was not identified using an adaptive change-point approach, which would make it difficult for generalization. Thirdly, there was no distinction among the various subtypes of migraine and the severity of migraine, which would be a direction to explore in future research. Furthermore, there was also no comparison between migraine and other primary headache disorders, and the distinction between migraine and other headache sufferers would be a direction that deserves attention. Finally, how to validate further clinically the models in practice by neurologists is also a question that needs to be considered.

## 5. Conclusions

In this study, we found that sFC strength and DFCP features in the resting state can be successfully used for the automatic identification of migraine and normal individuals. As far as we know, this was the first study to use DFCP features at the scale of whole-brain regions in the resting state and a combination of sFC and DFCP features for migraine classification. In the cross-validation process, we divided the data into training and test datasets, with the test datasets coming from the two normal control groups. In order to validate the classification effect more rationally, the test set was not involved in the selection of features but was used as a black box. To verify the reliability and repeatability of the algorithm, the second normal control group in this paper was not involved in the feature selection and classifier training process. The results showed that the classification model has good recognition ability.

## Figures and Tables

**Table 1:** Performance evaluation of classifier using different classification approaches for COBRE dataset.

Approach	Time Window	Accuracy Std Mean with 95% CI	Precision Std Mean with 95% CI	Recall Std Mean with 95% CI	Specificity Std Mean with 95% CI	F1 Std Mean with 95% CI
sFC Strength	Static	0.8653 0.0853 [0.8486 0.8821]	0.8726 0.0974 [0.8535 0.8917]	0.8686 0.1372 [0.8417 0.8955]	0.8633 0.1107 [0.8416 0.8850]	0.8628 0.0931 [0.8461 0.8795]
DFCP	12 s	0.9382 0.0769 [0.9231 0.9533]	0.9381 0.0955 [0.9194 0.9568]	0.9495 0.0866 [0.9325 0.9665]	0.9264 0.1197 [0.9030 0.9499]	0.9397 0.0724 [0.9246 0.9547]
	24 s	0.9552 0.0527 [0.9449 0.9655]	0.9420 0.0796 [0.9264 0.9576]	0.9790 0.0523 [0.9688 0.9893]	0.9314 0.0983 [0.9122 0.9507]	0.9575 0.0490 [0.9471 0.9678]
	36 s	0.9311 0.0768 [0.9161 0.9462]	0.9204 0.1001 [0.9008 0.9401]	0.9562 0.0931 [0.9379 0.9744]	0.9076 0.1198 [0.8841 0.9311]	0.9329 0.0765 [0.9179 0.9480]
	48 s	0.9039 0.0751 [0.8892 0.9186]	0.8877 0.1016 [0.8678 0.9076]	0.9426 0.0704 [0.9245 0.9607]	0.8650 0.0924 [0.8389 0.8911]	0.9085 0.1330 [0.8938 0.9232]
	60 s	0.8904 0.0867 [0.8734 0.9074]	0.8827 0.1137 [0.8604 0.9050]	0.9210 0.1058 [0.9002 0.9417]	0.8595 0.1517 [0.8298 0.8893]	0.8946 0.0831 [0.8776 0.9116]
sFC Strength + DFCP	12 s	0.9583 0.0620 [0.9461 0.9704]	0.9520 0.0818 [0.9360 0.9681]	0.9724 0.0732 [0.9580 0.9867]	0.9450 0.0955 [0.9263 0.9637]	0.9592 0.0619 [0.9471 0.9714]
	24 s	0.9681 0.0467 [0.9590 0.9773]	0.9541 0.0726 [0.9399 0.9684]	0.9914 0.0397 [0.9837 0.9992]	0.9450 0.0895 [0.9275 0.9625]	0.9703 0.0431 [0.9612 0.9795]
	36 s	0.9487 0.0604 [0.9368 0.9605]	0.9281 0.0876 [0.9110 0.9453]	0.9836 0.0471 [0.9743 0.9928]	0.9129 0.1101 [0.8913 0.9344]	0.9525 0.0551 [0.9406 0.9643]
	48 s	0.9210 0.0745 [0.9064 0.9356]	0.9006 0.1015 [0.8807 0.9205]	0.9621 0.0813 [0.9462 0.9781]	0.8802 0.1292 [0.8549 0.9056]	0.9255 0.0702 [0.9109 0.9401]
	60 s	0.9128 0.0889 [0.8954 0.9303]	0.9017 0.1152 [0.8791 0.9243]	0.9452 0.0930 [0.9270 0.9635]	0.8821 0.1471 [0.8533 0.9110]	0.9170 0.0826 [0.8996 0.9344]

**Table 2:** The *p*-value and significance of classification performance matrix along three approaches under different time window lengths, which obtained by two-sample *t*-test for COBRE dataset. ‘*’ indicates a significant difference between two approaches.

Time Window	Approach	Accuracy	Precision	Recall	Specificity	F1
12 s	sFC Strength vs. DFCP	6.7688 × 10^−8^ (*)	3.9109 × 10^−6^ (*)	1.0474 × 10^−5^ (*)	8.9016 × 10^−5^ (*)	4.3069 × 10^−8^ (*)
	sFC Strength vs. sFC Strength + DFCP	1.2212 × 10^−15^ (*)	3.8783 × 10^−10^ (*)	5.7846 × 10^−10^ (*)	1.0144 × 10^−8^ (*)	6.2172 × 10^−15^ (*)
	DFCP vs. sFC Strength + DFCP	0.0451 (*)	0.2502	0.0512	0.2120	0.0442 (*)
24 s	sFC Strength vs. DFCP	6.4948 × 10^−14^ (*)	3.0128 × 10^−7^ (*)	4.5752 × 10^−11^ (*)	1.1576 × 10^−5^ (*)	5.5733 × 10^−14^ (*)
	sFC Strength vs. sFC Strength + DFCP	0 (*)	5.2878 × 10^−10^ (*)	2.4203 × 10^−14^ (*)	8.3851 × 10^−8^ (*)	0 (*)
	DFCP vs. sFC Strength + DFCP	0.0839	0.2831	0.0373 (*)	0.3260	0.0642
36 s	sFC Strength vs. DFCP	4.7401 × 10^−8^ (*)	0.0012 (*)	1.1628 × 10^−6^ (*)	0.0109 (*)	3.3973 × 10^−8^ (*)
	sFC Strength vs. Strength + DFCP	9.4036 × 10^−14^ (*)	3.3847 × 10^−5^ (*)	9.1094 × 10^−13^ (*)	0.0020 (*)	1.4877 × 10^−14^ (*)
	DFCP vs. Strength + DFCP	0.0801	0.5772	0.0049 (*)	0.7554	0.0414(*)
48 s	sFC Strength vs. DFCP	0.0011 (*)	0.2599	2.5415 × 10^−5^ (*)	0.9190	2.3344 × 10^−4^ (*)
	sFC Strength vs. Strength + DFCP	1.9914E-07 (*)	0.0401 (*)	3.6740 × 10^−8^ (*)	0.2904	2.5007 × 10^−8^ (*)
	DFCP vs. Strength + DFCP	0.1000	0.3475	0.1220	0.3798	0.0853
60 s	sFC Strength vs. DFCP	0.0386 (*)	0.4697	0.0040 (*)	0.8317	0.0126(*)
	sFC Strength vs. sFC Strength + DFCP	5.4790 × 10^−5^ (*)	0.0430 (*)	2.4769 × 10^−6^ (*)	0.2768	7.4323 × 10^−68^ (*)
	DFCP vs. Strength + DFCP	0.0781	0.2727	0.0812	0.3206	0.0586

**Table 3:** (ROIs) * information for sFC strength feature.

ROI Number	Abbreviation	Anatomic and Modified Cytoarchitectonic Description
45	ORGL3	Brodmann area 11 (lateral area 11) in orbital gyrus of frontal lobe
46	ORGR3	Brodmann area 11 (lateral area 11) in orbital gyrus of frontal lobe
47	ORGL4	Brodmann area 11 (medial area 11) in orbital gyrus of frontal lobe
50	ORGR5	Brodmann area 13 in orbital gyrus of frontal lobe
101	ITGL7	Brodmann area 20 (caudoventral of area 20) in inferior temporal gyrus of temporal lobe
102	ITGR7	Brodmann area 20 (caudoventral of area 20) in inferior temporal gyrus of temporal lobe
164	INSR1	hypergranular insula in insular gyrus of insular lobe
170	INSR4	ventral dysgranular and granular insula in insular gyrus of insular lobe

* ROI descriptions and abbreviations were obtained from the Brainnetome Atlas [26].

**Table 4:** Correlation coefficients between time window length and classification performance metrics for COBRE dataset.

Approach	Accuracy	Precision	Recall	Specificity	F1
DFCP	−0.8845	−0.9412	−0.7011	−0.9349	−0.8773
sFC Strength + DFCP	−0.9136	−0.9383	−0.7280	−0.9438	−0.8997

## Data Availability

The data of migraineurs were obtained from Department of Neurology of Shanghai Jiao Tong University Affiliated Sixth People’s Hospital. According to the Regulations on Human Genetic Resources Management published by the Chinese government and to ensure the privacy of patients, these data are not available. The data of normal controls were obtained from a free public database which can be accessed at http://fcon_1000.projects.nitrc.org/fcpClassic/FcpTable.html on 5 December 2020 and at http://fcon_1000.projects.nitrc.org/indi/retro/cobre.html on 15 December 2020.

## Supplementary Materials

The following supporting information can be downloaded at: https://www.mdpi.com/article/10.3390/brainsci13040596/s1, Table S1: Performance evaluation of classifier using different classification approaches for ICBM dataset; Table S2: the *p*-value and significance of classification performance matrix along three approaches under different time window lengths, which obtained by two-sample *t*-test for ICBM dataset; Figure S1: The classification Accuracy, Precision, Specificity, Recall, and F1 of sFC strength, DFCP, and sFC strength + DFCP approaches between the migraine patients and normal control (NC) subjects (ICBM dataset).
