# Supplementary material for: Classification of Migraine Using Static Functional Connectivity Strength and Dynamic Functional Connectome Patterns: A Resting-State fMRI Study
Source: Brain Sci. 2023 Mar 31;13(4):596. doi: 10.3390/brainsci13040596 (PMC10137025; doi:10.3390/brainsci13040596)
Supplement: Supplementary file 1 [file brainsci-13-00596-s001.zip › brainsci-2257988-supplementary.pdf]

Supplementary Material

1 Supplementary Tables

Table S1. Performance evaluation of classifier using different classification approaches for ICBM dataset.

Approach	Time Window	Accuracy<br>Std<br>Mean with 95% CI	Precision<br>Std<br>Mean with 95% CI	Recall<br>Std<br>Mean with 95% CI	Specificity<br>Std<br>Mean with 95% CI	F1<br>Std<br>Mean with 95% CI
sFC Strength	Static	0.7593<br>0.1061<br>[0.7385 0.7801]	0.7264<br>0.1152<br>[0.7038 0.7490]	0. 8686<br>0.1372<br>[0.8417 0.8955]	0.6512<br>0.1809<br>[0.6157 0.6866]	0.7831<br>0.0982<br>[0.7623 0.8039]
DFCP	12s	0.8298<br>0.0931<br>[0.8116 0.8481]	0.7780<br>0.1083<br>[0.7567 0.7992]	0.9495<br>0.0866<br>[0.9325 0.9665]	0.7105<br>0.1656<br>[0.6780 0.7429]	0.8502<br>0.0795<br>[0.8319 0.8684]
	24s	0.8566<br>0.0905<br>[0.8389 0.8744]	0.8025<br>0.1144<br>[0.7801 0.8249]	0.9790<br>0.0523<br>[0.9688 0.9893]	0.7348<br>0.1800<br>[0.6995 0.7700]	0.8766<br>0.0713<br>[0.8589 0.8944]
	36s	0.8514<br>0.0903<br>[0.8337 0.8691]	0.8041<br>0.1079<br>[0.7829 0.8252]	0.9562<br>0.0931<br>[0.9379 0.9744]	0.7471<br>0.1674<br>[0.7143 0.7800]	0.8675<br>0.0793<br>[0.8498 0.8852]
	48s	0.8407<br>0.0980<br>[0.8214 0.8599]	0.7975<br>0.1179<br>[0.7744 0.8206]	0.9426<br>0.0924<br>[0.9245 0.9607]	0.7402<br>0.1736<br>[0.7062 0.7743]	0.8580<br>0.0844<br>[0.8388 0.8772]
	60s	0.8149<br>0.1019<br>[0.7949 0.8349]	0.7783<br>0.1264<br>[0.7535 0.8031]	0.9210<br>0.1058<br>[0.9002 0. 9417]	0.7102<br>0.1901<br>[0.6730 0.7475]	0.8350<br>0.0873<br>[0.8150 0.8550]
sFC Strength +DFCP	12s	0.8678<br>0.0843<br>[0.8512 0.8843]	0.8170<br>0.1072<br>[0.7960 0.8380]	0.9724<br>0.0732<br>[0.9580 0.9867]	0.7638<br>0.1582<br>[0.7328 0.7948]	0.8829<br>0.0726<br>[0.8664 0.8994]
	24s	0.8832<br>0.0859<br>[0.8663 0.9000]	0.8316<br>0.1166<br>[0.8088 0.8545]	0.9914<br>0.0397<br>[0.9837 0.9992]	0.7762<br>0.1750<br>[0.7419 0.8105]	0.8991<br>0.0688<br>[0.8823 0.9160]
	36s	0.8770<br>0.0914<br>[0.8590	0.8263<br>0.1173<br>[0.8034	0.9836<br>0.0471<br>[0.9743	0.7702<br>0.1735<br>[0.7362	0.8934<br>0.0740<br>[0.8754

	0.8949]	0.8493]	0.9928]	0.8042]	0.9113]
48s	0.8590	0.8143	0.9621	0.7562	0.8751
	0.0847	0.1126	0.0813	0.1705	0.0692
	[0.8425	[0.7923	[0.9462	[0.7228	[0.8585
	0.8757]	0.8364]	0.9781]	0.7896]	0.8917]
60s	0.8390	0.7971	0.9452	0.7319	0.8578
	0.0964	0.1211	0.0930	0.1817	0..819
	[0.8201	[0.7733	[0.9270	[0.6963	[0.8389
	0.8579]	0.8208]	0.9635]	0.7675]	0.8767]

Table S2. The p-value and significance of classification performance matrix along three approaches under different time window lengths, which obtained by two-sample T-test for ICBM dataset; '*' indicates a significant difference between two approaches.

Time Window	Approach	Accuracy	Precision	Recall	Specificity	F1
12s	sFC Strength VS DFCP	1.7941×10^{-6} (*)	0.0010 (*)	1.0474×10^{-5} (*)	0.0167 (*)	5.5654×10^{-7} (*)
	sFC Strength VS sFC Strength+DFCP	7.2642×10^{-13} (*)	2.9628×10^{-8} (*)	5.7846×10^{-10} (*)	3.1908×10^{-6} (*)	4.8705×10^{-13} (*)
	DFCP VS sFC Strength+DFCP	0.0023 (*)	0.0093 (*)	0.0512	0.0190 (*)	0.0022 (*)
24s	sFC Strength VS DFCP	2.5013×10^{-11} (*)	8.7161×10^{-6} (*)	4.5752×10^{-11} (*)	0.0011 (*)	2.4685×10^{-11} (*)
	sFC Strength VS sFC Strength+DFCP	1.5654×10^{-14} (*)	5.3721×10^{-9} (*)	2.4203×10^{-14} (*)	2.8875×10^{-6} (*)	5.5511×10^{-16} (*)
	DFCP VS sFC Strength+DFCP	0.0188 (*)	0.0578	0.0373 (*)	0.0685	0.0139 (*)
36s	sFC Strength VS DFCP	5.2555×10^{-9} (*)	6.4053×10^{-6} (*)	1.1628×10^{-6} (*)	3.4674×10^{-4} (*)	2.9506×10^{-9} (*)
	sFC Strength VS Strength+DFCP	6.3116×10^{-13} (*)	8.3983×10^{-8} (*)	9.1094×10^{-13} (*)	1.4048×10^{-5} (*)	4.6629×10^{-14} (*)
	DFCP VS Strength+DFCP	0.0494 (*)	0.1701	0.0049 (*)	0.3385	0.0180 (*)
48s	sFC Strength VS DFCP	1.2785×10^{-7} (*)	4.0979×10^{-5} (*)	2.5415×10^{-5} (*)	6.8011×10^{-4} (*)	7.5558×10^{-8} (*)
	sFC Strength VS Strength+DFCP	3.9468×10^{-13} (*)	6.4139×10^{-8} (*)	3.6740×10^{-8} (*)	1.2944×10^{-5} (*)	1.0825×10^{-13} (*)
	DFCP VS Strength+DFCP	0.1572	0.2981	0.1220	0.5226	0.1091
60	sFC Strength VS DFCP	1.7425×10^{-4} (*)	0.0021 (*)	0.0040 (*)	0.0219 (*)	1.3054×10^{-4} (*)
	sFC Strength VS sFC Strength+DFCP	1.2766×10^{-7} (*)	3.3286×10^{-5} (*)	2.4769×10^{-6} (*)	0.0013 (*)	2.9471×10^{-8} (*)
	DFCP VS Strength+DFCP	0.0963	0.3041	0.0812	0.4292	0.0651

2 Supplementary Figure

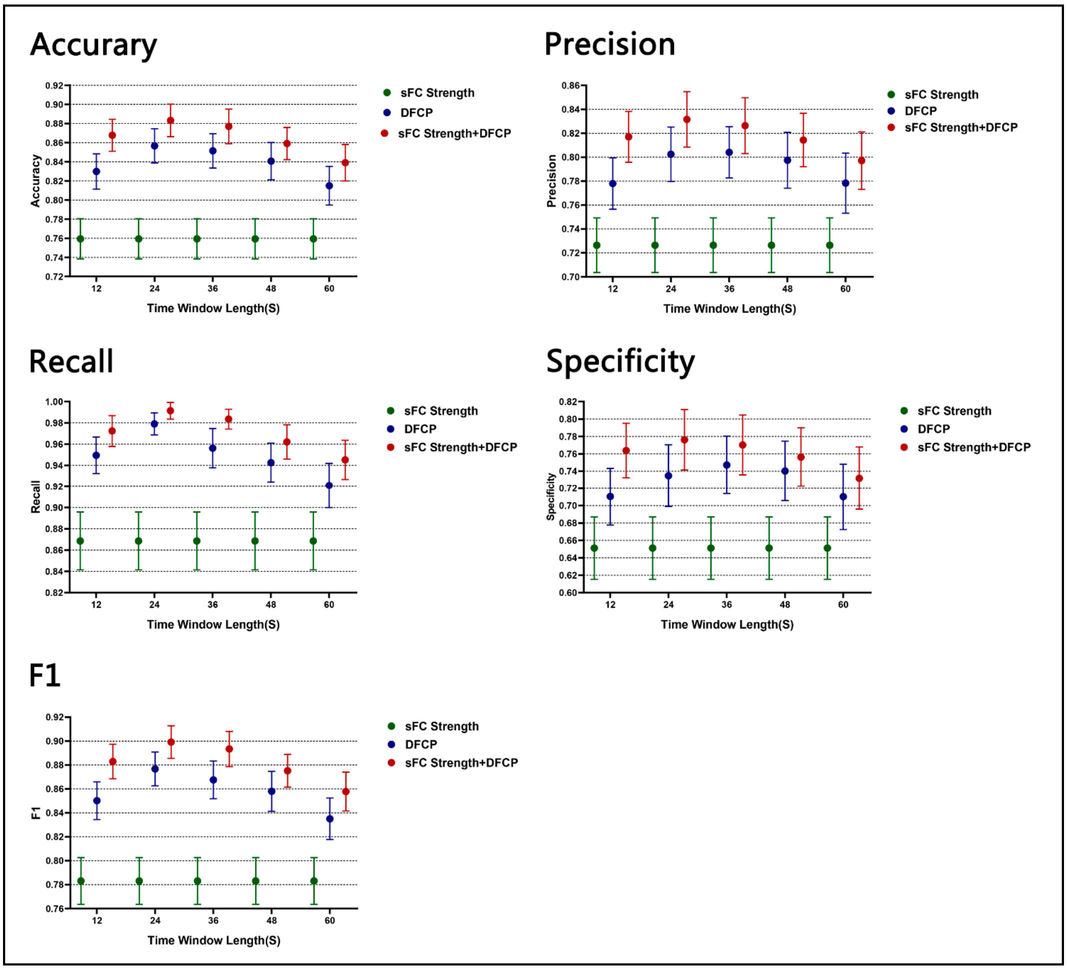

Figure S1. The classification Accuracy, Precision, Specificity, Recall, and F1 of sFC strength, DFCP, and sFC strength+DFCP approaches between the migraine patients and normal control (NC) subjects (ICBM dataset). On the X-axis, labels t = 12-60 indicate the time window length for DFCP, and Y-axis indicates the mean classification Accuracy, Precision, Recall, and F1 on CI 95%.

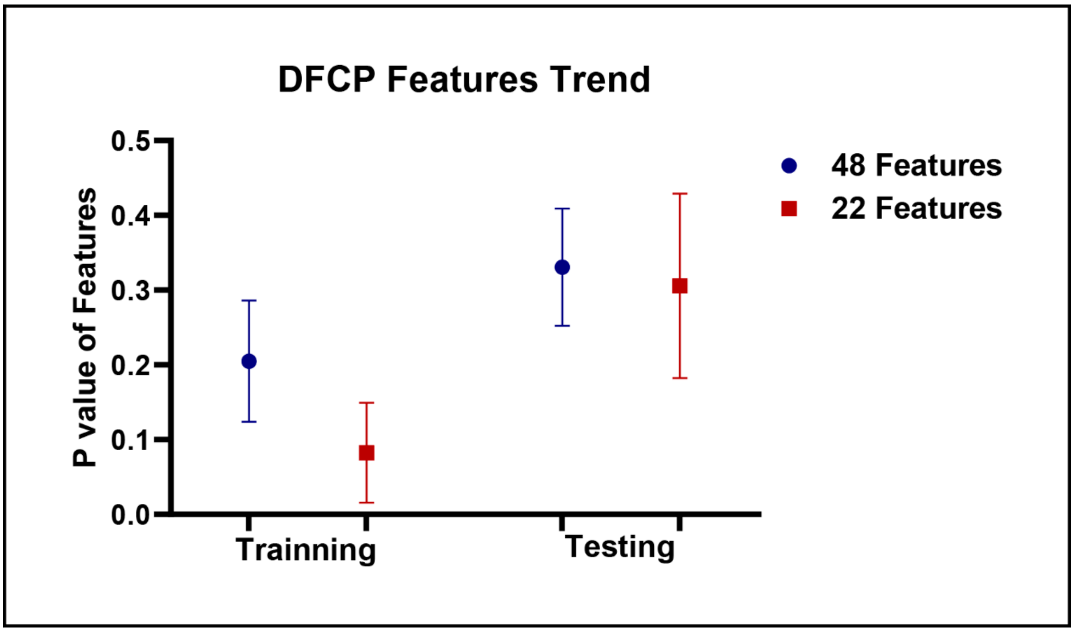

Figure S2. The Comparison of the mean P value for DFCP on CI 95%.
